# An Intervention to Increase Situational Awareness and the Culture of Mutual Care (Foco) and Its Effects During COVID-19 Pandemic: A Randomized Controlled Trial and Qualitative Analysis

**DOI:** 10.3389/fpsyt.2020.570786

**Published:** 2020-11-26

**Authors:** Elisa H. Kozasa, Shirley S. Lacerda, Monica Aparecida Polissici, Roseli da Silva Coelho, Gedeon da Silva Farias, Patrícia Chaves, Eliseth R. Leão

**Affiliations:** ^1^Hospital Israelita Albert Einstein, Instituto Do Cérebro, São Paulo, Brazil; ^2^Hospital Israelita Albert Einstein, São Paulo, Brazil

**Keywords:** situational awareness, mental health, health care workers (HCW), safety, care, mindfulness

## Abstract

Situational awareness is especially important to decision-making in health care. Comprehending the situation is crucial for anticipating any change in the environment and delivering optimal care. The objective of this study was to evaluate the effects of a training to increase situational awareness and mutual care designed for health care workers (FoCo) in a randomized controlled trial with additional qualitative analysis. We also investigated the perception of the training for the COVID-19 pandemic moment, in May 2020, almost 6 months after we finished the data collection at the Emergency Care Unit, which became a COVID-19 treatment reference for the care of a population depending on the public health system, in Sao Paulo, Brazil. We conclude that FoCo training can be an important instrument for health care professionals both in times of pandemic and “normal times,” to increase situational awareness, the culture of mutual care and decrease the possibility of occupational injuries and illnesses.

## Introduction

Situational awareness (SA) “describes the ability of an individual to maintain an adequate internal representation of the status of the environment in complex and dynamic domains where time constants are short and conditions may change within seconds and minutes” (1). It was initially studied in the context of aviation as a crucial factor for decision making. The failures in SA were classified in three levels: (I) -failure to correctly perceive the information, (II)- failure to comprehend the situation-, or (III) failure to project the situation into the future. In Level I, relevant data is not available; difficult to detect or discriminate; information is misperceived, or a memory problem happens. Level II involves mainly the use of an incorrect mental model, lack of or incomplete mental model, overreliance on default values. Level III involves, for example, an over projection of current trends (2). SA is especially important to decision-making in health care. Comprehending the situation to provide a mental model is crucial for anticipating any change in the environment and delivering optimal care (3).

It is known that stress is associated with health problems such as mental and cardiovascular disorders, also being a cause of absenteeism and reduced productivity in companies (4, 5). An increase in the risk of injuries and illnesses at work has been associated with fatigue, stress, haste, distraction, emergency situations, excessive noise, complex procedures and anger, among other factors (6). A large part of these factors decreases attention from work in progress and may affect SA. This situation can be improved by training workers' attention and awareness during daily activities, since it would encourage a return to focus on the task at hand and the possible risks associated with it. Participants in a mindfulness training group significantly improved the aspect of attention known as *orienting* when compared to participants in a control group (7). In a review of brain regions and mindfulness training, the authors proposed the involvement of the anterior cingulate and striatum (attention control), prefrontal and limbic regions and striatum (emotional regulation), insula, medial prefrontal cortex and posterior cingulate cortex and precuneus (self-awareness) (8). In other words, mindfulness training presents repercussions not only in cognitive, but also in emotional control, and can be one of the factors which improves SA.

In the health care area, studies conducted with nursing teams suggest that mindfulness can be an effective and inexpensive way to reduce symptoms of anxiety, depression, psychological distress and burnout, improve well-being, increase quality of life and a provide a higher level of satisfaction with life (9–11).

SA is important for the safety of patient and health care team with improved clinical outcomes. The environment is complex and dynamic especially when high technology is involved, and complex human interactions change from moment to moment. Human factors are core in SA, therefore any team member should challenge another without fear. Mutual care demands that the team fell free to speak up if they notice anything which may compromise the quality of care or safety of team members (3).

On 25th May 2020, as a result of the COVID-19 pandemic, there were 5,370,375 confirmed cases in 216 countries and 344,454 confirmed deaths. Consequently, health care professionals are facing the most challenging situation in this century (12). In a meta-analysis about the mental health of health care workers (HCW), anxiety presented a prevalence of 23.2%, insomnia 38.9% and depression 22.8%. Female HCW and nurses showed higher rates of affective symptoms compared to male and medical staff. Training SA abilities became vital to ensure the safety and quality of care of HCW.

In 2019, before any sign of COVID-19 pandemic, an intervention to increase SA and mutual care (Focus con Consciousness—FoCo) was developed and tested in two units, an Emergency Care Unit and a Residential Facility for the Elderly in Sao Paulo, Brazil. As far as we know, after a search in PubMed, there were no other studies with similar interventions for health care professionals evaluated before and during an epidemic or pandemic. We hypothesized that FoCo would increase mindfulness, self-compassion, positive affect, sleep quality, and reduce stress perception, negative affect, and symptoms of mental diseases.

The objective of this study was to evaluate the effects of a training to increase situational awareness and mutual care designed for HCW in their working hours. We also investigated the perception of the FoCo training for the COVID-19 pandemic moment, in May 2020, almost 6 months after we finished the data collection at the Emergency Care Unit, which became a COVID-19 treatment reference for the care of a population depending on the public health system, in Sao Paulo, Brazil.

## Materials and Methods

### Participants

**Figure 1:**
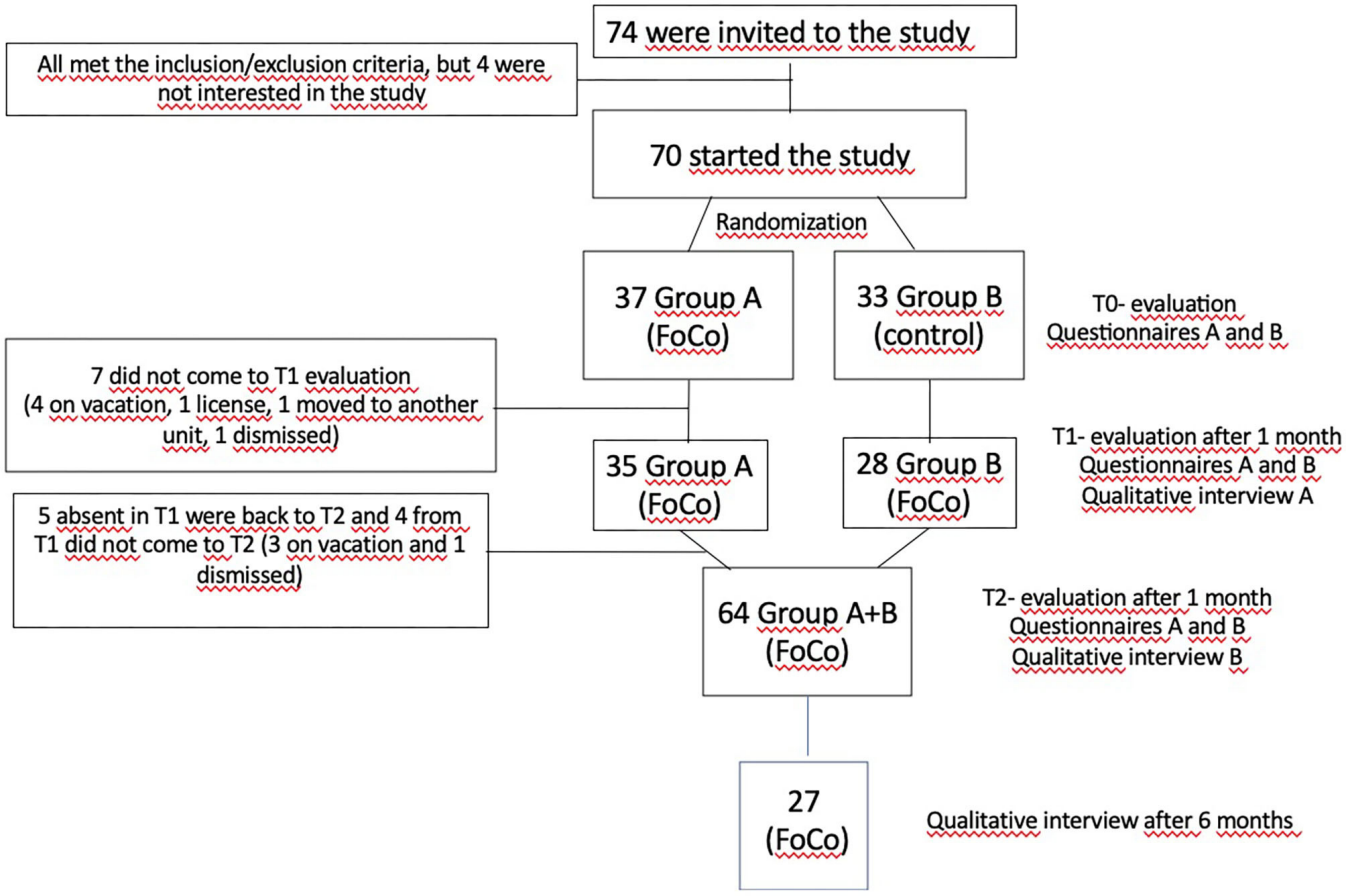
Flow chart of the study.

Health care professionals (nurses, nursing technicians, physical therapists and caregivers of the elderly) of both genders, ages from 18 to 60 years old, working in two units, 41 from an Emergency Care Unit (ECU) and 29 from a Residential Facility for the Elderly (RE), participated in the study. All 74 professionals from the ECU and RE working during daytime where invited to participate in the study (Figure 1).

Inclusion criteria: being available to participate in the training and carry out evaluations before and after it.

Exclusion criteria: being away or on vacation in the research project period, or under psychiatric treatment.

This study is registered in clinicaltrials.gov NCT04362397 and has the Ethical Committee approval number 14788419.1.0000.0071.

### Questionnaire and scales

- Socio-demographic and clinical data: data that characterize the participants in the socioeconomic, educational, mental and physical health status.
- Mindful Awareness Attention Scale (MAAS): Using scales from 1 to 6, the individual classifies how he/she experiences everyday situations, answering 15 questions about their level of attention or awareness (13).
- Self-Compassion Scale (SCS). This is a 26-item scale that measures how someone typically acts toward themselves in difficult times. The subject has to choose from 1 to 6 points on a Likert scale (14).
- Positive and Negative Affect Scale (PANAS): scale composed of a list of 20 affects (10 positive and 10 negative) in which the participants respond on five-point Likert scales (15).
- Self-Report Questionnaire (SQR-20): An inventory for the detection of psychiatric symptoms with 20 questions about mental health (16).
- Perceived stress scale (PSS) This scale measures the degree to which individuals perceive situations as stressful. The items were designed to assess how unpredictable, uncontrollable, and overburdened the people evaluated consider their lives. PSS is a general scale and can be used in different age groups, as it does not contain specific contextual issues. It is composed of 10 items related to sensations (17).
- Pittsburgh Sleep Quality Index (PSQI): this scale with 19 questions assesses the sleep quality of the interviewee in the last 30 days, through objective questions (18).

### Procedure

In T0, 74 participants were invited to the study and 70 agree to participate. The professionals were randomized in groups A and B in the second semester of 2019. From T0 to T1, 7 participants did not come to T1 evaluation (four on vacation, one license, one moved to another unit, one dismissed); five participants absent in T1 were back to T2 and four from T1 did not come to T2 (three on vacation and one dismissed) (more details in Figure 1–flow chart).

The researcher responsible for the statistical analysis randomized the participants inside each professional category (i.e., nurses, nursing technicians, physical therapists and caregivers of the elderly) generating a list of random numbers in excel and were blind to the intervention and control groups. Group A received training in the FoCo program and group B comprised a waiting list group. After a month, group B received the training and group A was encouraged to continue the intervention. Participants were assessed before intervention and 1 month thereafter therefore, it was not possible to blind participants to the groups. A follow-up was scheduled during the COVID-19 pandemic to evaluate its effects.

During all the study the safety department of the hospital were monitoring the participants to investigate possible harms or adverse effects.

#### FoCo Intervention

Initially the leaders of the health care departments attended a 1-h training and started to practice the intervention. The health care professionals, under the guidance of their leaders, were invited to participate in the study. They were categorized according to their profession and randomized to group A or B within their category. After signing the informed consent and filling the questionnaires, group A received the training, which lasted only 10–15 min.

The content of the training is based on the three pillars of FoCo:

- Becoming Aware (BA): everyday, before starting the routine, the participants in small groups closed their eyes and payed attention to their breathing; performed a relaxation exercise; remembered the main tasks of the day, that is, to be gentle with themselves and others; to speak clearly to the colleagues and patients, and to cultivate the inner and outer well-being. Additionally, they could practice Becoming Aware at any time of the day, including at home with their families.
- License to Care (LC): if the participant noticed that a colleague had forgotten to put on any equipment of personal protection, or perform an important procedure, he or she was to advise immediately; if the participant met someone during the routine who did not look well, whether a patient or a colleague, he or she was expected to ask if the person needed any help.
- Observation and Behavioral Approach (OBA): an observer who is trained in OBA would approach a professional and tell him or her if they agreed to be observed during their routine tasks. If they agreed, the observer would stay there for a while and after that would give constructive feedback about their behavior, especially focused in safety. This approach was already implemented in the institution before the entire FoCo intervention.

#### Statistical Analysis

The data collected were analyzed using the JASP program. Categorical data were expressed as percentage and the frequency distributions between groups were analyzed using Chi-square tests. For numerical data, analyses were carried out regarding the distribution of data, whereas for data with parametric distribution, the *t*-test was used, and for those with non-parametric distribution, the Wilcoxon test. Subsequently, Analysis of Variance for repeated measures were used to evaluate the effects of the intervention over time between groups. Pearson correlation and Linear Regression analyses were also performed.

#### Qualitative Analysis

Thirteen participants of different professional categories (nurses, nursing technicians, physical therapists, and caregivers of the elderly) from the ECU and RE, representative of the sample recruited to the study, with a good narrative, were invited for interviews. Participants of both groups, A and B, were included in these interviews 1 month after receiving the FoCo training. For the 1 month interview, at least one representative of each professional category was selected, those who had a good narrative and communication skills to enrich the qualitative assessment. The interviews ended when finding theoretical data saturation by through the identification of the absence of new elements in each thematic group.

The interviews were recorded, transcribed and submitted to content analysis as described by Bardin (19). The most emblematic speeches of each category were presented in the results. The full content analysis report is presented as Supplementary Materials 1, 2.

#### Guiding Questions

- What is your perception of the training in Becoming Aware?
- What is your perception of the License to Care? How do you feel about undergoing Behavioral Observation and Approach training?
- How do you perceive your attention and awareness regarding your work activities in the last month?

For the 6 months interviews, during the COVID-19 pandemics all the 27 professionals who were at the Emergency Care Unit on the day of the interviewer's visit participated in the evaluation. We would like to have included the questionnaires and scales in this evaluation, however, due to the pandemic, the ICU professionals had a short time to answer questions and were dressed with all personal protective equipment's, therefore the interviewer were allowed only to record the interviews.

Questions asked to the Emergency Care Unit health care professionals during COVID-19 (almost 6 months after we finished the FoCo study in 2019):

- What is your perception of FoCo training, in which you participated, regarding this moment of COVID-19?
- What training did you use during the pandemic? And in what situations?

## Results

There were no differences between ECU and RE in the questionnaires at baseline them, therefore we decided to perform the statistical analysis grouping the units. Groups A and B there showed no differences in gender, age, years of education, socioeconomic status and mental health, neither regarding psychiatric symptoms, self-compassion, mindfulness, perceived stress, positive and negative affect and sleep quality at baseline (Table 1). The safety department of the hospital which was monitoring the participants during all months of the study did not detect any harm or adverse effect due to the FoCo intervention.

**Table 1:** Comparison between groups at baseline.

	**Group A (*n* = 37)**	**Group B (*n* = 33)**	**Sig**.
Age Mean (sd)	33.30 (8.08)	35.94 (9.19)	0.205^t^
Gender *n* (%)			
Male	9 (24.32%)	4 (12.12%)	0.190^c^
Female	28 (75.68%)	29 (87.88%)	
Years of Schooling *n* (%)			
<8	1 (2.70%)	1 (3.03%)	0.739^c^
9–11	14 (37.84%)	10 (30.30%)	
12–16	11 (29.73%)	12 (36.36%)	
17 or more	11 (29.73%)	10 (30.30%)	
Ethnicity *n* (%)			
Caucasian	13 (35.14%)	10 (30.30%)	0.123^c^
African-Brazilian	8 (21.62%)	2 (6.06%)	
Brown	15 (40.54%)	21 (63.34%)	
Asian	1 (2.70%)	0 (0%)	
Marital Status *n* (%)			
Single	11 (29.73%)	14 (42.42%)	0.098^c^
Married	16 (43.24%)	9 (27.27%)	
Lives together	9 (24.32%)	4 (12.12%)	
Divorced	1 (2.70%)	4 (12.12%)	
Widowed	0 (0%)	2 (6.06%)	
Religion *n* (%)			
Atheist	1 (2.70%)	0 (0%)	0.558^c^
Without religion	4 (10.81%)	7 (21.21%)	
Catholic	15 (40.54%)	11 (33.33%)	
Protestant	2 (5.40%)	0 (0%)	
Evangelic	12 (32.43%)	12 (36.36%)	
Spiritualist	1 (2.70%)	2 (6.06%)	
Other	2 (5.40%)	1 (3.03%)	
Monthly income (minimum wages) *n* (%)			
<1	1 (2.70%)	3 (9.09%)	0.426^c^
1–3	14 (37.84%)	10 (30.30%)	
3–5	12 (32.43%)	10 (30.30%)	
5–8	10 (27.03%)	8 (24.24%)	
8 or more	0 (0%)	2 (6.06%)	
SRQ	3.41 (3.05)	2.85 (2.81)	0.431^t^
SCS	88.65 (17.44)	93.18 (11.56)	0.210^t^
MAAS	70.97 (9.73)	72.39 (10.08)	0.551^t^
PSS	15.97 (6.42)	16.68 (5.77)	0.638^t^
PANASP	33.32 (6.03)	34.97 (5.88)	0.253^t^
PANASN	15.24 (3.96)	15.36 (4.68)	0.908^t^
PSQI	6.16 (3.40)	6.18 (3.62)	0.981^t^

t Student t test;

c *Chi-squared test. SRQ, Self-Report Questionnaire; SCS, Self-Compassion Scale; MAAS, Mindful Attention Awareness Scale; PSS, Perceived Stress Scale; PANAS-P, Positive and Negative Affect Schedule—Positive Affects; PANAS-N, Positive and Negative Affect Schedule—Negative Affects; PSQI,Pittsburgh Sleep Quality Index*.

In the comparison of both groups before and after 1 month of intervention in group A, both groups improved concerning psychiatric symptoms, self-compassion, and perceived stress (Table 2). After 1-month evaluation, group B also received the intervention. Since both groups improved from baseline to 1 month similarly, we decided to cluster A and B in one group in the comparison of the training from baseline to 2 months, when we found improvements in perceived stress and quality of sleep (Table 3).

**Table 2:** Comparison between baseline and 1 month.

	**Group A (*****n*** **=** **35)**		**Group B (*****n*** **=** **28)**		**Time effect**	**Group effect**	Time*Group effect
	**Baseline**	**1 month**	**Baseline**	**1 month**			
	**Mean (s.d.)**	**Mean (s.d.)**	**Mean (s.d.)**	**Mean (s.d.)**			
SRQ	3.31 (3.11)	2.46 (2.78)	3.04 (2.99)	2.61 (3.41)	0.031^*^	0.929	0.466
SCS	88.60 (15.61)	95.46 (16.43)	93.89 (11.01)	96.79 (13.65)	0.007^**^	0.313	0.257
MAAS	71.09 (9.99)	72.03 (11.09)	72.18 (10.35)	71.71 (10.95)	0.803	0.877	0.464
PSS	16.09 (6.48)	13.80 (5.87)	16.68 (5.61)	14.93 (5.65)	0.008^**^	0.515	0.718
PANAS-P	33.60 (5.99)	34.23 (6.81)	34.82 (6.21)	35.22 (6.68)	0.438	0.467	0.868
PANAS-N	15.26 (4.01)	13.69 (4.09)	15.30 (3.94)	15.48 (6.33)	0.260	0.369	0.155
PSQI	6.03 (3.43)	5.71 (3.15)	6.29 (3.67)	6.25 (3.49)	0.584	0.625	0.663

* p < 0.05;

** *p < 0.01. SRQ, Self-Report Questionnaire; SCS, Self-Compassion Scale; MAAS, Mindful Attention Awareness Scale; PSS, Perceived Stress Scale; PANAS-P, Positive and Negative Affect Schedule—Positive Affects; PANAS-N, Positive and Negative Affect Schedule—Negative Affects; PSQI, Pittsburgh Sleep Quality Index*.

**Table 3:** Comparison between baseline and 2 months.

	**All participants (*****n*** **=** **64)**		**Time effect**
	**Baseline**	**2 months**	
	**Mean (s.d.)**	**Mean (s.d.)**	
SRQ	2.83 (2.75)	2.43 (3.09)	0.231
SCS	90.25 (14.63)	93.41 (16.94)	0.095
MAAS	72.31 (9.91)	73.47 (12.24)	0.246
PSS	16.13 (6.14)	13.92 (6.69)	0.004^**^
PANAS-P	34.25 (5.90)	34.14 (7.67)	0.900
PANAS-N	15.31 (4.40)	15.23 (6.40)	0.909
PSQI	5.83 (3.23)	5.02 (3.20)	0.048^*^

* p < 0.05;

** *p < 0.01. SRQ, Self-Report Questionnaire; SCS, Self-Compassion Scale; MAAS, Mindful Attention Awareness Scale; PSS, Perceived Stress Scale; PANAS-P, Positive and Negative Affect Schedule—Positive Affects; PANAS-N, Positive and Negative Affect Schedule—Negative Affects; PSQI, Pittsburgh Sleep Quality Index*.

As PSS showed significant differences from baseline to 1 month and 2 months, we decided to explore a correlation analysis. PSS is correlated with all variables at baseline (*p* < 0.05), except PANASP. Therefore, to evaluate the effect of MAAS, SCS, SRQ, PANAS-N, and PSQI on PSS, multiple linear regression models were built using the stepwise method. The linear regression model was significant for the variables MAAS, SRQ and PANAS-N (*p* < 0.001), with the SRQ (ß = 0.676; *p* < 0.001). PANAS-N (ß = 0.560; *p* = 0.007) proved to be positive predictors for PSS, while MAAS (ß = −0.124; *p* = 0.033) was a negative predictor for PSS (Table 4).

**Table 4:** Linear regression model for PSS as the dependent variable.

***R*^**2**^**	***F* (3,66)**	***p***	**Covariates**	**ß**	**SE**	***t***	***p***
0.540	25.794	<0.001	PANAS-N	0.676	0.131	5.153	<0.001^**^
			SRQ	0.560	0.202	2.776	0.007^**^
			MAAS	−0.124	0.057	−2.175	0.033^*^

R^2^, 0.540;

* p < 0.05;

** *p < 0.01. PSS, Perceived Stress Scale; SRQ, Self-Report Questionnaire; MAAS, Mindful Attention Awareness Scale; PANAS-N, Positive and Negative Affect Schedule—Negative Affects*.

Qualitative analysis revealed the following categories after 1 month of training FoCo (detailed reports are in Supplementary Material 1). HCW of groups A and B participated in the interviews:

1. **Becoming Aware**
  - **Greater Self-Awareness**
    - Ah! I think this perception that we sometimes do things automatically (RIAE01)
    - … made us aware, because sometimes you are so stressed, and you stop, you breathe, you think, you relax, you do the exercise, then you come back to yourself and you can continue (UPA37)
    - It is sometimes you already come a little stressed from the street, I don't know, maybe because I was stuck in traffic. And then you arrive, you already find a patient already nervous. Then the patient's nervousness added to that stress, right?… it's time for you to stop, make that reflection and become aware… (UPA15)
  - **Situational Awareness**
    b.1) **Greater attention to tasks**
      - … before doing the procedure, I already plan in my head what I have to do, and then at the time of execution I can do everything calmly, without that rush,… take a breath and go! (RIAE15)
      - … I think it increased my attention on tasks, I also tried to leave the phone in silent mode, to not even vibrate, because I realized that when it vibrated, I was distracted, so. when I am going to do some activity that I know I need more attention I… leave it silent. (RIAE01)
      - … now we start paying more attention, right, with the question of putting a medication, or a movement that you do wrong, it all can harm, so we start to pay more attention (UPA25)
      - … we are able to focus on exactly what we are doing there at the moment, what you have to think, what you think later, the important thing is that moment you are doing something (UPA25)
    b.2) **Identification of risks and errors**
      - … sometimes you break that paradigm, everyone doing the same thing wrong, sometimes a simple thing,… why doesn't someone always do that part at this moment? (UPA37)
      - sometimes we are on the run, right, the person doesn't look sometimes, forgets to put on a PPE, then you warn, calm down, let's put on the PPE first, let's wear the glasses, the gloves, so I always think about orientation and aiming… (UPA40)
      - Especially in relation to the use of alcohol gel, before and after,… we are not doing it, these are small things that make a lot, a lot of difference, right? (UPA15)
    b.3) **Decision making**
      - so we didn't make a decision when feeling angry, we used it (the training) even on a daily basis. (UPA25)
      - and helped me like that, in decision-making actually… In the stress of everyday life, even during patients screening, when something happened… before making a certain rash decision, I used this technique. (UPA35)
    b.4) **Attitude**
      - … when you do it, you turn on this “key,” and then you propose to yourself… become aware of what you are doing, of your actions. let's assume a moment of aggression, a moment of the patient's nervousness, you kind of prepare both to respond, and to embrace this patient, you know, embracing… it gets easier. (UPA08)
      - Using the techniques, with the guidelines, I found that I can develop my work better,… you can calm down, have the correct behavior for that situation, so I thought it was very important, that it was… was good. (UPA35)
  - **Well-Being Doing “Becoming Aware”**
    - … we come back relaxed, do something good like that, you come back with another disposition, with spirit and it's being very good (RIAE48)
    - … I, I felt relaxed like this, I felt at peace, I felt like I don't know, it's invigorating (UPA37)
    - I'm less stressed because of the awareness, less stressed. (UPA09)
2. **License to Care**
  - **Self-Care**
    - why the patient always comes first and sometimes we put ourselves last too… so this awareness-raising course was for us to see ourselves, as a professional, as a person (RIAE19)
    - try to be generous, with us I have an extremely difficult time with this… and then, be generous with myself,… wow, I needed this. (RIAE01)
    - Sometimes you are not cool or sometimes, I don't know, or there is a patient… destabilized you and you went out of focus a little bit, go to the bathroom and breathe and come back more, you come back more relaxed, to give. that breath? That unburdened… you get a little out of that focus so you can go to that “Becoming Aware,” you come back lighter, you come back calmer. (UPA15)
  - **Caring For The Other**
    - that we have to pay attention from day to day and have more, have attention, I think for the care, not only for us, but also for our patients. (RIAE48)
    - This possibility of helping the colleague, right? Often the person is in a moment of stress, is going through some kind of problem and he can't even… and you have this freedom to get to him and… and guide, know if you're going through some kind of problem,… this really happened during the shift with a colleague… I believe it helped her a lot… (UPA35)
    - The person is there learning, is nervous… and… you arrive and pass a security there to the colleague, right? Transmitting security there to him… and you can arrive and pass it on to your colleague safely… (UPA15)
  - **License to Care is Gratifying**
    - … I think that being able to help the patient in need, being able to do a little for them, I think this is very rewarding for us. (RIAE48)
    - It was done, that was the feeling. Why, as much as the person doesn't want to talk, at least you're willing, and even if she doesn't want to talk at that moment, she knows she can come back and talk… and sometimes our colleague just needs one, a support, someone to hear what you want to say, that's good! (RIAE19)
    - ahhhhh, it's gratifying right? You can help a colleague who is going through a certain type of problem, sometimes that person cannot see that she is going through this. (UPA35)
  - **Incomprehension about the Training License to Care**
    - … at no point during the training of the focus did I notice a freedom to do this with the people who were around me and there was also nothing specific for that. (RIAE01)
    - License to care? (UPA-44)
    - I think there could be more training in this… license to take care, maybe some way that we could be trained to do it … in a kind way with people, (RIAE01)
3. **Post-end Analysis of Training**
  - **A New Habit**
    - I think it's really important to make a habit of it, to make it something more automatic to do every day. (RIAE01)
    - Look, I have done it, sometimes with our manager here, and I have also done it sometimes at home (RIAE15)
    - I used it on several days, right, when you get caught in the same routine, in the daily rush, then I caught myself, stopping, doing my 5 min there, breathing and becoming aware of the daily planning. (UPA40)
  - **Extension of the Practice to Other Places**
    - at your home, with your friends, your family, … you arrive, you arrive a little more stressed, because it is not easy, right, the hospital environment is not easy, but then you arrive a little more stressed, then you don't wait, let me breathe, let me center, let me breathe, inhale (UPA37)
    - … I used it, both in my work environment, as it was proposed, and also before I get home I do it too. (UPA08)
    - … what I tried to pass on to them from what I learned, … although they are small eight and 12 (kids), they have a certain awareness, so the 12 1 day he said “I'll do it too.”(RIAE12)
  - **Positive Evaluation of the Focus Project**
    - I wanted to say that I liked it a lot, it's very good, whoever has the opportunity to do it, has to do it, because it helped me, and I'm sure it helped many colleagues who did it from the beginning, (UPA25)
    - Ah! I found it super interesting, because that training gave us an awareness of how we get centralized, of how you get back to the axis, (UPA37)
    - I believe that nothing is in vain, you know, that everything is to improve, it is if this was proposed to us, growth, you know, individual growth, of the group and finally, of the department. (UPA08)

Almost 6 months after receiving FoCo training, 27 participants of the study belonging to the Emergency Care Unit, which became a COVID-19 treatment reference, taking care of a population that depends on the public health system, were interviewed again, for us to investigate their perception of the FoCo training for the COVID-19 pandemic moment. They had to change their working schedule from daily 6 h (their original one) to 12-h shifts (1 day at work, 1 day off) when the pandemic started, and the unit was full of patients. During one of the shifts, which concentrated most of the participants of last year's study, we performed the interviews. One of the participants received COVID-19 treatment and presented post-traumatic stress disorder symptoms and was not able to answer the questions. Another one we had to exclude from the study because she reported that she had answered the questionnaires but had not received the training directly from the instructor. Therefore, we had 25 valid interviewees (detailed reports are in Supplementary Material 2). The following categories were identified:

1. **The Impact of Covid-19**
  - I was leaving my house with tachycardia… arriving here sincerely with a pain in the belly, you know, just thinking that I was going to a place, … where everything was going to be COVID (UCL08)
  - it's a lot of intubation, the whole routine has changed (UCL24)
  - there are many moments when we feel exhausted… there are many moments that we don't seem to be able to… that will not work… we think about giving up …
  because it is not easy! (UCL16)
  - we are afraid to take the coronavirus to our family (UCL36)
2. **Importance of FoCo at this Time of COVID-19**
  - **To myself**

It seems that they were predicting something (the pandemic) … giving the FoCo training… we even comment about it in all shifts… how important it is to be maintaining the emotional psychological balance, So I see… the FoCo training is extremely important at this moment (UCL29)

- At the time it was done (the training) we didn't really give it much importance, even though we thought it would be important. Today we see here that it really felt right for us to do it (UCL32)
- I think it is extremely important due to the stress that we are going through in our daily routine. (UCL39)

I think it was important just as we are treating a patient with COVID, it affects us a lot, our psychological, I think it was important for us to work better on this side of us, from the nervousness of the fear of being contaminated, we have to work even with emotions (UCL34)

Wow !!! in my point of view, especially at this moment, it is being well-used, I believe not only for me, but a lot for the other colleagues who are using this method…(UCL16)

b) **To the work**
  - At this moment when the demand is greater, we are managing to concentrate more on what we are doing, on the patients that are reaching …the most critical patients, so we are managing to have the perception and this focus on the patient (UCL20)
  - …and we were going to receive these patients… and it's been very useful for me, it's helping a lot in my day to day, in my work! (UCL08)
  - It is what I am managing to do: to take a step backwards in the face of the chaos that sets at times. So, I have to leave the scene and try to visualize what is happening from the outside in order to enter and have a different attitude. (UCL27)
  - …try to stay calm, pay attention to what I'm doing, the procedures (UCL34)
**3) Becoming Aware to Recover Balance**
  - …because we go through several moments during the shift … when you really have to stop, go somewhere, take a breath and get balanced and come back to the fight again. (UCL29)

I do it several times a day… there is some extreme moment when I need to stop 5 min, breathe, drink water, think about what is important now, because there are many things happening at the same time and we end up losing track. (UCL30)

- … before dealing with the most critical patients intubated… stopping, breathing, trying to keep calm in order to continue (UCL39)
- Ah sometimes the stress… emotional stress, right? Sometimes I get a little more reclusive, I try to leave, I've done this a few times, I leave the place, it's quieter, I sit for about 5 min waiting a little bit, and then I can put the ideas in place, then I'll be back again (UCL10)

Right now it's being important because the unit is very full there… when we stop, take a deep breath, I at least understand why I am here, what is my importance at this moment, I feel I feel calmer to be able to perform my duties.

(UCL36)

So at this moment, especially at this moment, it's being really cool for us to use… we need to stop to repeat breathing and come back, right at a point that we can start again, but more peaceful…, that's where you stop, breathe, think, get a grip of yourself and come back! That is exactly what I have been doing many times. I have needed to make the “Becoming Aware.”(UCL16)

Sometimes we faced situations, then you remember “Becoming Aware” and you take a step away, breathe, …wait, be calm (UCL07).

**4) Integrating Self-Care With the Care for Others**
  - …because I often perceive a sad colleague, crying, crestfallen and then I usually try to get him, get him out of the sector a bit, go up to the kitchen to have water, have coffee… so I think that in addition to taking care of myself I'm looking at the next one too (UCL29)

I particularly wear all the PPE that is available and I try to wear it in the best possible way because apart from the patients I care for here, I have a family that I have to care for at home… we have an …openness among all colleagues to be able to call attention, showing what is wrong … we talk regardless of the level of education, no matter if you are a technician. we always have an opening with everyone. (UCL32)

I wear PPE all the time, I went into the shock room or in the observation room I already have glasses on, my mask I don't take off even to drink water, and gloves are on all the time … yes if I see a colleague at risk of infection without wearing glasses or a mask I always give an alert (UCL09).

- My perception was that my care increased, … both with me and with the patient to whom I provide this care (UCL14)
- …after we took the course so we can pay more attention to help colleagues… raise awareness of colleagues … we see something that may cause some risk and raising awareness to try to keep calm… (UCL34)
- I think the care of the team in general is three times more than before, right, because of the fear of contamination, of contaminating a colleague, of contaminating oneself, of not taking it home, I think it was having an impact… you know, it ends up generating self-care, you have to be careful with others, whoever is there with you then automatically because of everything we have been through in this training, in the past, I think it fits well (this moment) (UCL10)
- we are always alerting the next colleague, right… what I don't want for myself I don't want him to go through, right? (UCL43)
- We know that we have to take care of the other, but before taking care of the other??? (UCL24)
**5) Someone Taking Care of Myself**

The “License to Care” … I find it extremely important because I often feel someone is taking care of me (UCL29)

Yes, there was even a day when I was not emotionally well, you know, and one of the people noticed, he gave me a helping hand, so he called me, we talked a lot, I managed to keep my shift very calm, you know… it had an effect. (UCL10)

yes, in fact I ended up receiving this guidance from my colleagues, because I ended up being contaminated with COVID-19, so I ended up receiving more than passing. (UCL18)

**6) Caring for Others**

I observe the colleagues and whenever possible I do it: to remind the colleague to do it (the practice) (UCL08)

we are watching each other a lot … helping the other …giving a nudge at the other, like… come here colleague let's … stop, … sometimes the colleague in that rush is stressed and you get there, “wait a minute, stay a little bit here… I go there to help the colleague to do the work … so he can take a breath … we are helping each other a lot! (UCL04)

I have used the care I do the OAC (Observation and Behavioral Approach) I have applied with colleagues. (UCL19)

it is at the moment when I saw a colleague, right, having a difficulty, I called him, asked him if he needed anything, if everything was fine with him, and I had that moment of conversation. I believe it improved the situation he was experiencing at the time! (UCL17)

we end up looking more at the team, right? I'll look more closely at people's behavior, … realizing who is not well, I call to talk,… but we end up helping each other more (UCL07)

**7) Difficulty in Applying “Becoming Aware,” in Spite of Realizing the Importance of It**
  - I think it would be excellent if we were able to actually apply it because … it is very busy due to the demand … we do not have time to apply the training, but I remember that when I did it, it was very relaxing, very wonderful (UCL19)
  - …it is during working hours I haven't been able to do it, but before leaving home I always stop for a while, I think, I try to breathe, calm down in order to have a more peaceful journey (UCL34)
  - … in the beginning I practiced a lot, it was really good, it works, but now at this moment it's so busy, that there's no time for us to stop a little bit, …now there's no time for even drinking water, I practiced a lot but now it's all over, but it was very good. (UCL15)
**8) FoCo for Life**
  - I would say that I even became a better person, because from the moment you take things more clearly, more calmly, you become a better person, so it has helped a lot, not only at work, in life. (UCL24)
  - My perception is that it helps a lot in our life, I even put it here in my room I always do it in the morning before leaving home (UCL07)

## Discussion

The aim of this study was to evaluate the effects of a short training to increase situational awareness and mutual care designed for HCW. We also investigated the perception of this training for the COVID-19 pandemic moment, in May 2020, almost 6 months after we finished the data collection at the Emergency Care Unit which became a COVID-19 treatment reference.

Before and after 1 month, both groups had improved in psychiatric symptoms, perceived stress and self-compassion (Table 2). Group A received the intervention and practiced 5 days a week with their leaders during this period of time. Group B participants belonged to the same health care units and despite having not received the FoCo training, they worked under the same leadership (who received the training before the research intervention started), with the colleagues of group A and possibly having some kind of interaction about the training with them. We instructed group A to avoid sharing the FoCo intervention with group B, however it could have happened. It may explain why group B also improved in their symptoms. Although we expected differences between groups, the literature has shown the impact of leadership on the work environment (20). Leadership can affect the workforce as well as the delivery of healthcare. In an article about nursing leaders, they are identified as playing an important role in retention of nurses, producing quality outcomes for staff nurses and patients, therefore affecting the whole health care environment (21). In a review about social and behavioral science to give support to COVID response, the authors mention the importance of leaders as representative members of the team (22). In FoCo, leaders practice with the team and this can be one of the explanations for its effectiveness. Improved psychiatric symptoms, perceived stress and self-compassion are also in line with the qualitative analysis, in which participants reported increased well-being doing “Becoming Aware” (feeling calm, relaxed, less stressed), greater self-awareness (increased perception and ability to deal with nervousness, tension) and self-care (doing well to take care of others, being generous to themselves) and an increase in risk perception.

After 2 months (groups A and B clustered in one), the evaluation results indicated improvements in perceived stress and quality of sleep (Table 3). Stress reduction seems to be the main response to the intervention, lasting from 1 to 2 months after baseline evaluation. In the linear regression model, SRQ and PANAS-N proved to be positive predictors for PSS, and MAAS was a negative predictor for PSS. MAAS is the scale to measure mindfulness. Mindfulness is a component of SA and classically related to stress reduction and associated variables such as cardiovascular improvement and sleep (23–25). It is also an important factor for mental health, and moderates the effects of perceived stress on depression, emotional exhaustion, anxiety, positive affect and negative affect (26). On the other hand, there were no differences in the scores of mindfulness along the study. The absence of differences in MAAS scores could be explained by the high scores in this scale of the HCW who participated in the study. Before the intervention, the mean score was 71.6 ± 9.85. In the validation study of MAAS in a Brazilian sample, the mean was 64.44 ± 7.73 in a group of meditators and 61.77 ± 10.48 in a sample of non-meditators (27). In another study evaluating a group of participants who have been practicing meditation for more than 1 year, their mean before a meditation retreat was 55.7 ± 11.08 and after that 63.6 ± 8.27 (28). An explanation for the high scores of mindfulness of this sample is the high level of training in this ability by health care professionals. The ECU and the RE are administered by the 38o. best hospital in the world and receive high quality training to deal with the daily activities (29). Despite MAAS scores having not changed through the study, qualitative analysis suggested increased perception of self-awareness, attention to the tasks, and decision making. In this analysis, just after 1 month of FoCo training, the categories related to awareness were more emphasized, which means were more frequently mentioned by the interviewees.

Increasing SA and mutual care (the aims of FoCo) allows participants to feel supported by colleagues, as we can see in qualitative analysis in the categories “license to care is gratifying,” “caring for others,” “identification of risks and errors” and “well-being doing Becoming Aware,” “greater self-awareness” and reduced stress symptoms.

Some participants reported difficulty to implement “License to Care,” not feeling comfortable to talk to the colleagues to “correct” their behavior or ask whether they needed help. This is a feedback that we gave to the units involved in the project and the others where FoCo was implemented to reinforce the importance of “License to Care” training. Risk perception workshops in addition to Leaders talks and BA reinforcement sections were part of the “License to Care” augmentation process.

COVID-19 pandemic is really challenging for the clinical staff. In a study comparing administrative and clinical staff, fear, anxiety and depression were significantly increased in the latter. Front line medical staff, including working in the critical departments such as respiratory, emergency, infectious disease, and ICU, showed higher scores on the scale of fear, anxiety and depression (30). The interviews for the data collection, performed at the ECU (a reference in treatment of COVID-19) on a day when it was full of patients, showed the importance of the training before the pandemic and its incorporation into the routine.

It is interesting that all participants from the 25 valid interviews have been using FoCo training during the pandemic, some of them doing part of it such as “License to Care,” and many of them keeping it a regular practice. Participants recognized the importance of this previous training last year for enabling them to deal with the stress of the pandemic, giving them the capacity to calm down when necessary, utilizing “Becoming Aware” and “License to Care.” The initial difficulty reported to apply “License to Care” last year, that is, taking care of the colleagues, especially related to equipment of personal protection use, and being able to offer support when someone was probably not feeling well, was not present during the pandemic. The team was really committed to taking care of themselves and the group. The category “Integrating self-care and the care of other” appeared during the pandemic, and the reports emphasized the mutual care that they were exercising at the ECU. Other changes reported were the reduction of hierarchal gradients, so that everyone at the HCW would feel comfortable to challenge each other without fear of consequences; mutual respect within the members, assuring the mutual care and everybody's right to speak up, if they noticed anything wrong or inefficient. It prevented turning a blind eye, improved patient and team safety (3). Basically, they were grateful for having received the training last year and understood that it promoted the culture of mutual care and increased situational awareness.

There are other hospitals implementing mental health protection and well-being promotion interventions for HCW (31). However, to the best of our knowledge, this is the first study that evaluates the effects of an intervention and its effects during the present pandemic. This study has limitations such as the control group working in the same environment of the intervention group and having leaders who were already trained in the intervention. A future study should guarantee that the control group work in another unit with similar characteristics. Nevertheless, the randomized control study with additional qualitative analysis brought a wealth of information, and we recommend keeping this approach in further projects.

Considering this combined approach, we conclude that FoCo training can be an important instrument for health care professionals both in times of pandemic and “normal times,” to increase situational awareness, the culture of mutual care and decrease the possibility of occupational injuries and illnesses.

## Data Availability Statement

The original contributions generated for this study are included in the article/Supplementary Materials, further inquiries can be directed to the corresponding author/s.

## Ethics Statement

The studies involving human participants were reviewed and approved by Ethics committee of the Hospital Israelita Albert Einstein. The patients/participants provided their written informed consent to participate in this study.

## Author Contributions

EK: designed the study, analyzed the data, wrote the manuscript, and revised the manuscript. SL: analyzed the data and revised the manuscript. MP, RC, and GF: collected data and revised the manuscript. PC: designed the study and revised the manuscript. EL: analyzed qualitative data and revised the manuscript. All authors contributed to the article and approved the submitted version.

## Conflict of Interest

The authors declare that the research was conducted in the absence of any commercial or financial relationships that could be construed as a potential conflict of interest.
