# Supplementary material for: An Intervention to Increase Situational Awareness and the Culture of Mutual Care (Foco) and Its Effects During COVID-19 Pandemic: A Randomized Controlled Trial and Qualitative Analysis
Source: Front Psychiatry. 2020 Nov 26;11:570786. doi: 10.3389/fpsyt.2020.570786 (PMC7725753; doi:10.3389/fpsyt.2020.570786)
Supplement: Supplementary file 2 [file Data_Sheet_2.docx]

**CATEGORIES**

**13 PARTICIPANTS - ONLY HEALTHCARE PROFESSIONALS**

**1) BECOMING AWARE**

**a) GREATER SELF-AWARENESS**

- ... as you are physically, psychologically, and then you prepare for the day with more tranquility, ... you breathe, you can do everything more calmly. (RIAE15)

~~- we can see when we are leaving a pattern that had started ... to be calmer. (RIAE19)~~

~~-... for you to really see your physical conditions, ... psychological, ... even spiritual (RIAE19)~~

~~- ... "wow, I got home" and that was a shock to me, ... mainly driving so as not to cause any accidents. (RIAE01)~~

~~- ... I think I'm more focused, ... I breathe, I'm more of a person here, so now I breathe, sometimes I even count the breaths (UPA37)~~

-... before judging the other, you observe yourself, see your behavior ... (UPA08)

- Ah I think this perception that we sometimes do things automatically (RIAE01)

- ... getting nervous just gets in the way, so I took a breath, made myself aware and said: “it will work” and I went, and for me it was good thanks to God ... (UPA25)

- ... made us aware, because sometimes you are so stressed, and you stop, you breathe, you think, you relax, you do the exercise, then you come back to yourself and you can continue (UPA37)

~~- ... I think it's a focus on our health, ... on our psychological, to see if we don't have ... a problem, or something like a depression, something like that. (RIAE48)~~

-It is sometimes you already come a little stressed from the street, I don't know, maybe because I was closed in traffic. And then you arrive, you already find a patient already nervous. Then the patient's nervousness added to that stress, right? ... it's time for you to stop, make that reflection and become aware ... (UPA15)

**b) SITUATIONAL AWARENESS**

**b.1) Greater attention to tasks**

- ... before doing the procedure, I already plan in my head, what I have to do, and then at the time of execution I can do everything calmly, without that rush, ... take a breath and go! (RIAE15)

- ... I think it increased my attention on tasks, I also tried to leave the phone in silent mode to not even vibrate, because I realized that when it vibrated, I distracted, so ... when I am going to do some activity that I know I need more attention I... leave it silent. (RIAE01)

~~- ... stop and pay attention, have a better awareness of what I'm doing and what the person wants to tell me. (RIAE01)~~

- ... we start paying attention to things that we don't pay on the run (UPA25)

- ... "man" I'm able to pay attention to things I didn't pay before (UPA25)

- ... now we start paying more attention, right, with the question of putting a medication, or a movement that you do wrong, it can all harm, so we start to pay more attention (UPA25)

- ... we are able to focus on exactly what we are doing there at the moment, what you have to think, what you think later, the important thing is that moment you are doing (UPA25)

~~- ... For us to be more focused on what, in our activity at that moment, always seek the focus, right, what we're doing, attention. (UPA09)~~

- My attention is much higher, I have stopped more, sometimes you are doing it in a rush, you know, in a hurry, there ... "don't wait there, let me look again, let me breathe" ... (RIAE19)

it ended up helping me ... to remember the tasks I had for the day and not to forget things that were essential for the task, because before I forgot a lot. ~~Ah I felt that I ended up forgetting less things during the day, I was more attentive to what I was going to do and in a way it helped like that~~ (RIAE01)

-... yeah, suddenly things that I don't realize I'm doing, not in the wrong way, but I could be doing it better, my colleague can observe this, then he gives me this feedback. (UPA29)

~~- ... so I think it is essential even for the professional, you know, for professionalism it was really useful, to have awareness and execution of the day ... of planning, ... so it was really effective. (UPA40)~~

**b.2) Identification of risks and errors**

~~-... and then she was without glasses and at the time ... it burst and the blood came right, and then I told her for the love of God, don't forget to wear glasses, (UPA25)~~

-... sometimes you break that paradigm, everyone doing the same thing wrong, sometimes a simple thing, ... why doesn't someone always do that part at this moment? (UPA37)

~~- So you can even put this on for yourself, you know, tell us how I did it, so now I realize that I may also be making mistakes right now. (UPA37)~~

~~- I realized that people are more attentive, more focused on the use of PPE (personal protection equipment), and it only took me a day, to pick up and talk to my colleague. (UPA09)~~

- Because she went to puncture the patient without the glasses, then I took it and said like that, hey… don't forget to wear the glasses, see! (UPA09)

- sometimes we are on the run, right, the person doesn’t look sometimes, forget to put on a PPE, then you warn, calm down, let's put on the PPE first, let's use the glasses, the gloves, so I always think about orientation and aiming... (UPA40)

. Especially in relation to the use of alcohol gel, before and after, ... we are leaving it, these are small things that make a lot, a lot of difference, right? (UPA15)

~~- ... I pay more attention to what I'm doing, talking, my tasks, PPE's, (RIAE01)~~

**b.3) Decision making**

- so we didn’t make a decision when feeling angry, we used it (the training) even on a daily basis. (UPA25)

- and helped me like that, in decision-making actually ... In the stress of everyday life, even during patients screening, when something happened ... before making a certain rash decision, I used this technique. (UPA35)

**b.4) Attitude**

~~- ... brings awareness of your activities, your attitude, you know, the way you really have to act, you have to do it is really cool (RIAE12)~~

-... when you do it, you turn on this "key", and then you propose to yourself ... become aware of what you are doing, of your actions ... let's assume a moment of aggression, a moment of the patient's nervousness, you kind of prepare both to respond, and to embrace this patient, you know, embracing ... it gets easier. (UPA08)

- Using the techniques, with the guidelines, I found that, I can develop my work better, ... you can calm down, take the correct behavior, for that situation, so I thought it was very important, that it was .. . was good. (UPA35)

**c) WELL-BEING DOING “BECOMING AWARE”**

- ... we come back relaxed, do something good like that, you come back with another disposition, with spirit and it's being very good (RIAE48)

-... even to relax, sleep, it helps a lot (UPA25)

-... I, I felt relaxed like this, I felt at peace, I felt like I don't know, it's invigorated (UPA37)

- Look, it made a big difference, every day it made a difference, because I got up, did it at home before going to the school and already makes me lighter. (UPA09)

~~- Less stressed, more focused, right, more attentive, that's it~~

- I'm less stressed because of the awareness, less stressed. (UPA09)

~~- ahhh, I like it… it was much more relaxing, it was much more, the procedure to the patient was more relaxed, you know? (UPA15)~~

- I realized that in addition I was influencing my, perhaps my mood, during the day, to be calmer, more calm to perform the tasks and know that I will be able to do it, there is no use running. (RIAE01)

I got a lot better, ... I got a little calmer, a little calmer (UPA35)

**2) LICENSE TO CARE**

**a) SELF-CARE**

~~-... we must be doing well to be able to take care of others, it is that we take care, but we end up forgetting to take care ... of us ... we end up leaving it aside. (RIAE48)~~

- why the patient always comes first and sometimes we put us last too ... so this awareness-raising course was for us to see ourselves, as a professional, as a person (RIAE19)

~~- ... I'm really tired, so ... you don't take from yourself what you don't have, so ... I really took a shower ... I rested, that's when I got better. (RIAE12)~~

- try to be generous, with us I have an extremely difficult time with this ... and then, be generous with myself, ... wow, I needed this. (RIAE01)

-Sometimes you are not cool or sometimes, I don't know, or there a patient ... destabilized you and you went out of focus a little bit, go to the bathroom and breathe and come back more, you come back more relaxed, to give ... that breath? That unburdened. ... you get a little out of that focus so you can go to that “Becoming Aware”, you come back lighter, you come back calmer. (UPA15)

**b) CARING FOR THE OTHER**

~~- a friend who was ... going through some difficulties at work, and then he told me that he … hadn't had lunch that he didn't want to have lunch, then I bought food and took it to his house, so I felt helping someone like this. (RIAE01)~~

- that we have to pay attention from day to day and have more, have attention, I think for the care, not only of us, but also of our patients. (RIAE48)

- This possibility of helping the colleague, right? Often the person is in a moment of stress, is going through some kind of problem and he can’t even ... and you have this freedom to get to him and… and guide, know if you’re going through some kind of problem, ... this really happened during the shift with a colleague ... I believe it helped her a lot ... (UPA35)

- The person is there learning, is nervous ... and ... you arrive and pass a security there to the colleague, right? Transmitting security there to him ... and you can arrive and pass it on to your colleague safely ... (UPA15)

**d) LICENSE TO CARE IS GRATIFYING**

- Well, it was really cool even because I think there was some feedback within what I was able to advise and talk about, I saw it later, a feedback from my colleague saying what I talked to her, the advice I spoke to her helped a lot. .. (RIAE12)

- ... I think that being able to help the patient in need, being able to do a little for them, I think this is very rewarding for us. (RIAE48)

- It was done, that was the feeling. Why, as much as the person doesn't want to talk, at least you're willing, and even if she doesn't want to talk at that moment, she knows she can come back and talk ... and sometimes our colleague just needs one , a support, someone to hear what you want to say, that's good! (RIAE19)

- I like it, I already like to take care naturally, so with the license, it was much more rewarding (UPA25)

~~-I felt very good, because of what he gave me, the answer he gave me, you see, he really could improve, you know, (UPA08)~~

- ahhhhh, it's gratifying right? You can help a colleague who is going through a certain type of problem, sometimes that person cannot see that she is going through this. (UPA35)

**e) INCOMPREHENSION ABOUT THE TRAINING THE LICENSE TO CARE**

- ... at no point during the training of the focus, I noticed a freedom to do this with the people who were around me and there was also nothing specific for that. (RIAE01)

- License to care? (UPA-44)

- Yes, but I didn't feel confortable doing it (RIAE01)

- I think there could be more training in this ... license to take care, maybe some way that we could be trained to do it … in a kind way with people, (RIAE01)

**3. POST-END ANALYSIS OF TRAINING**

**3.a) THE NEED TO BECOME A NEW HABIT**

- I think it's really important to make it a habit to become something more automatic to do every day. (RIAE01)

- Yes, sometimes I did it at home, before going to school, before exams at school, I took a breath, that we are very anxious, so it is good to have these 5 minutes is good, yes. (RI)

- Look, I have done it, sometimes with our manager here, and I have also done it sometimes at home (RIAE15)

~~- we have to have a moment for us, the moment, for 5 minutes, to understand, I think it should be daily, this, always, but most of the time we end up scamming and only when we are really very tense is that we do (RIAE19)~~

- I think what remains to be cooler is to do this regularly. (RIAE19)

- suddenly get up and do it and especially at night, it became the time to relax, right? (RIAE12)

- I used several days, right, when you get caught in the same routine, in the daily rush, then I caught myself, stopping, doing my five minutes there, breathing and becoming aware of the daily planning. (UPA40)

- Always do this every day ... I did it many days. I don't know if they were all consecutive (they weren't all consecutive), but I did a lot. But it was very important, I felt much better. (UPA15)

~~- In the beginning, I had difficulty concentrating, just not thinking about anything and realizing myself, that was the biggest difficulty, (RIAE19)~~

**3.b. EXTENSION OF THE PRACTICE TO OTHER PLACES**

- ... I did it myself at various times, at home and here during the shift.

 (UPA29)

- It was very different, because I'm sure that if I hadn't met this project, I would get nervous, ... it's a change that I wanted ... I dealt with it very differently in several situations after the project (UPA29)

- at your home, with your friends, your family, … you arrive, you arrive a little more stressed, because it is not easy, right, the hospital environment is not easy, but then you arrive a little more stressed, then you don't wait, let me breathe, let me center, let me breathe, inhale, ~~and then you can come back…~~ (UPA37)

~~- …it's because I used it out here, too. (UPA09)~~

-... I used it, both in my work environment, as it was proposed, and also before I get home I do it too. (UPA08)

-... what I tried to pass on to them from what I learned, … although they are small 8 and 12 (kids) have a certain conscience, so the 12 until one day he said "I'll do it too". (RIAE12)

**~~3.d IMPROVE PROJECT DISSEMINATION~~**

~~I think the groups could have been publicized, ... I often didn't know about the training. (RIAE01)~~

**3.e POSITIVE EVALUATION OF THE FOCUS PROJECT**

~~- In my perception, it was a very good training ... (UPA25)~~

- I wanted to say that I liked it a lot, it’s very good, whoever must have the opportunity to do it, has to do it, because it helped me, and I’m sure it helped many colleagues who did it from the beginning, (UPA25)

~~- Yes, I found an interesting project, because it deals directly with the employee and his way of life, right (UPA29)~~

- Ah! I found it super interesting, because that training gave us an awareness of how we get centralized, of how you get back to the axis, (UPA37)

- ... I did find it interesting, because it opens our gaze right, as a whole, both with a colleague and with ourselves. (UPA09)

- I believe that nothing is in vain, you know, that everything is to improve, it is if this was proposed to us, growth, you know, individual growth, of the group and finally, of the department. (UPA08)

~~- well the training reached the goals with me, right ... (...) it was very meaningful (UPA40)~~

- it was very intuitive, right, very constructive, this whole process, it was really useful, it was really effective. (UPA40)

~~- I thought so, the experience was very good, right ... I think it really helped a lot. (UPA35)~~

- I was satisfied ... (RIAE01)

- ... and that has helped a lot in the day-to-day. (RIAE48)

~~- very nice, very good. (RIAE19)~~
