# Supplementary material for: An Intervention to Increase Situational Awareness and the Culture of Mutual Care (Foco) and Its Effects During COVID-19 Pandemic: A Randomized Controlled Trial and Qualitative Analysis
Source: Front Psychiatry. 2020 Nov 26;11:570786. doi: 10.3389/fpsyt.2020.570786 (PMC7725753; doi:10.3389/fpsyt.2020.570786)
Supplement: Supplementary file 3 [file Data_Sheet_3.docx]

**1) THE IMPACT OF COVID-19**

-I was leaving my house with tachycardia ... arriving here sincerely with a pain in the belly, you know, just thinking that I was going to a place, … where everything was going to be COVID (UCL08)

-it's a lot of intubation, the whole routine has changed (UCL24)

- there are many moments when we feel saturated ... there are many moments that we don't seem to be able to… that will not work… we think about giving up …

because it is not easy! (UCL16)

-we are afraid to take the coronavirus for our family (UCL36)

**2) IMPORTANCE OF THE FoCo AT THIS TIME OF COVID-19**

**2.1 To myself**

It seems that they were predicting something so ... we even comment on all shifts about it ... how important it is to be maintaining the emotional psychological balance, So I see ... the FoCo training is extremely important at this moment (UCL29)

~~-for us it was very useful because we had to stop, right, rethink, breathe, so we could serve our patient. So, I have used it in all situations because we have to be focused to better serve our clientele and also stop and think ... (UCL31)~~

~~-It is now more than ever, right, it is being, it is very effective … (UCL08~~)

-At the time it was done (the training) we didn't really give it much importance, even though we thought it would be important. Today we see here that it really fell right for us to do it (UCL32)

-I think it is extremely important due to the stress that we are going through daily routine. (UCL39)

~~-This moment is extremely important because there are more difficulties ... the employee is now a little more concerned with the situation and is more subject to some types of problems. (UCL17)~~

I think it was important just as we are treating a patient with COVID, it affects us a lot, our psychological, I think it was important for us to work better on this side of us, from the nervousness of the fear of being contaminated, we have to work even with emotions (UCL34)

~~It was important, right, because everything is focused on the psychological, physical, so this training that we had previously is helping a lot for us not to focus so much on the problem that is happening, not to affect the emotional (UCL03)~~

~~My perception is very good, I think it helped us a lot, otherwise everyone would be much more nervous than they are now, which kind of doesn't know how to deal with the situation, so I think it helped a lot! (UCL24)~~

Wow !!! in my point of view, especially at this moment, it is being well used, I believe not only for me, but a lot for the other colleagues are using this method, at this moment we are seeing me because it is difficult. (UCL16)

~~... sometimes you have to stop, make yourself aware, breathe, do the whole process, and it helps a lot because it is organized in the school's organizational psychology at work, I think it's really cool, yes it is working!~~

~~(UCL07)~~

**2.1. To the work**

-It is really this FoCo training ... it comes in handy, this is a moment that we really need to focus on the actions that we have to perform ... (UCL30)

-At this moment when the demand is greater, we are managing to concentrate more on what we are doing, on the patients that are reaching …the most critical patients, so we are managing to have the perception and this focus on the patient (UCL20)

-It was being used more, sometimes it ended up going unnoticed, previously forgotten, but now at COVID it’s really super important to do it every day (UCL35)

and we were going to receive these patients ... and it's been very useful for me, it's helping a lot in my day to day, in my work! (UCL08)

-It is what I am managing to do: to take a step backwards in the face of the chaos that sets in at times. So I have to leave the scene and try to visualize what is happening from the outside in order to enter and have a different attitude. (UCL27)

- …try to stay calm pay attention to what I'm doing, the procedures (UCL34)

**3) BECOMING AWARE TO RECOVER BALANCE**

because we go through several moments during the shift … when you really have to stop, go somewhere, take a breath and get balanced and come back to the fight again. (UCL29)

~~I used the part we use a lot, think about what we're doing, breathe, reflect and go back to our work, because we end up doing everything mechanically, with risk for the patient, risk of exposure to the virus, so a moment concentration and breathing and stopping those 5 min before starting any action is of utmost importance!~~ I do it several times a day… there is some extreme moment that I need to stop 5 min breath, take a water, think about what is important now, because there are many things happening at the same time and we end up losing track. (UCL30)

~~Very important, especially when it comes to stopping, taking a deep breath, you know how to try to relax and start over again, because it is very stressful so like without comments right! It was very good, I liked it a lot, and we are enjoying what we learned there. (UCL04)~~

-… before dealing with the most critical patients intubated… stopping, breathing, trying to keep calm in order to continue (UCL39)

-Ah sometimes the stress... emotional stress, right? Sometimes I get a little more reclusive, I try to leave, I’ve done this a few times, I leave the place, it’s quieter, I sit for about 5 minutes waiting a little bit, and then I can put the ideas in place, then I'll be back again (UCL10)

~~I believe it helps a lot because, whether we like it or not, we get a little overwhelmed, tension, you know, caring for patients at risk of contaminating us, and also contaminating our family members, so it is a moment that before entering the shift we stop, think, take a deep breath and come on (UCL43)~~

Right now it's being important because the unit is very full there ... when we stop, take a deep breath, I at least understand why I am here, what is my importance at this moment, I feel I feel calmer to be able to perform my duties.

(UCL36)

~~we are managing to take it well, so we can manage it well, so I think that awareness has helped, because there was a moment that gives a little fear but then you stop, you analyze, breathe and calmly take care of yourself first and then taking care of the other (UCL24)~~

So at this moment, especially at this moment, it's being really cool for us to use ... we need to stop to repeat breathing and come back, right at a point that we can start again, but more peaceful ..., that's where you stop , breathe, think, compose yourself and come back! That is exactly what I have been doing many times. I have needed to make the “Becoming Aware”. (UCL16)

~~I am using the moment of reflection a lot in this pandemic so that of you breathing, breathing for 5 minutes there, to resume, to do some activity with the patient, well for me, it is helping a lot.~~

~~(UCL18)~~

~~I always do (pauses, not complete awareness) so straightforward, with a patient who takes us seriously, we go there breathe, really focus and it works.~~

~~(UCL15)~~

~~The awareness of you stopping a certain moment of stress, in fact I even did it now at that moment, someone actually approached me, in fact I was a little busy, approached me asked me to keep calm, a certain balance to give continuity on duty. (UCL35)~~

Sometimes we faced situations, then you remember “Becoming Aware”and you take a step away, breathe, …wait, be calm (UCL07).

**4) INTEGRATING SELF-CARE WITH THE CARE OF OTHERS**

-…because I often perceive a sad colleague, crying, crestfallen and then I usually try to get him, get him out of the sector a bit, go up to the kitchen to have a water, have a coffee ... so I think that in addition to taking care of myself I'm looking at the next one too (UCL29)

~~we have to take care of us and the colleague, sometimes the colleague is in a situation and we have to get him out of that situation so as not to get infected ... we have to realize and then and act quickly so that he don't get contaminated ... (UCL31)~~

I particularly use all the PPE that is available and I try to use it in the best possible way because apart from the patients I care for here, I have a family that I have to care for at home. I use all that are available. Look at our team, we have a …openness among all colleagues to be able to call attention, showing what is wrong … we talk regardless of the level of education, no matter if you are a technician. we always have an opening with everyone. (UCL32)

I wear PPE all the time, I went into the shock room or in the observation room I already have glasses on my mask I don’t take the time off even to drink water and gloves all the time (on leave to take care)… yes if I see a colleague in risk of infection without wearing glasses or a mask I always give an alert (UCL09).

~~yes, I had already observed that colleagues doing, right, in relation to wearing glasses, not touching sense without us sitting with the glove, so I saw some colleagues doing this and this is this collegiality, it ends up being more present, so I see it more!~~

~~(care culture) Taking care of oneself, right? (UCL27)~~

-My perception was that my care increased, … both with me and with the patient for whom I provide this care (UCL14)

-…after we took the course so we can pay more attention to help colleagues… raise awareness of colleagues … we see something that may cause some risk and raising awareness to try to keep calm… (UCL34 )

~~Now in the pandemic, when I see any situation that I will be very upset, I go to the bathroom, I take a deep breath, I try to focus on my professional and focus here, I am here to help them! (UCL03)~~

-I think the care of the team in general is 3 times more than before, right, because of the fear of contamination of contaminating a colleague, of contaminating oneself, of not taking it home, I think it was having an impact... you know, it ends up generating self-care, you have to be careful with others, whoever is there with you then automatically because of everything we have been through in this training, in the past, I think it fits well (to this moment) (UCL10)

-we are always alerting the next colleague, right ... what I don't want for myself I don't want him to pass through, right? (UCL43)

-We know that we have to take care of the other, but before taking care of the other, ~~it is watching us, taking care of ourselves, (UCL24)~~

~~especially in this moment that we are living we have done this a lot, it is always paying attention to the colleague, the other, it is and whenever necessary we call, we give advice, you know, the more attentive the person is, the better for everyone right, so take care of your mind, take care of your own health, also take care of your colleague's health. (UCL16)~~

-you end up coming back from lunch and someone tells you: “look at your glasses”! … it's being a two-way thing, in fact, I'm also correcting ... guiding and also receiving guidance…certainly everything that was applied in the training is helping a lot, it's great. (UCL35)

**5) SOMEONE TAKING CARE OF MYSELF**

The “License to care”… I find it extremely important because I often feel someone taken care of me (UCL29)

Yes, there was even a day when I was not emotionally well, you know, and one of the people noticed, he gave me a helping hand, so he called me, we talked a lot, I managed to keep my shift very calm, you know ... it had an effect. (UCL10)

yes, in fact I ended up receiving this guidance from my colleagues, because I ended up being contaminated with COVID-19, so I ended up receiving more than passing. ~~Receiving a lot of messages even a little earlier, because I was referring today, I’m not cool, I’m not 100%, I think you better talk, seek a doctor to undergo an evaluation, always in this sense, this guidance.~~ (UCL18)

**6) CARING FOR OTHERS**

I observe the colleagues and whenever possible I do it: to remind the colleague to do it (the practice) ~~you know, when we end up sitting in the place to have lunch at lunchtime you see the faces of the colleagues right and then you think, reflect and we talk about it.~~ (UCL08)

we are watching each other a lot … helping the other …giving a nudge at the other, like… come here colleague let's … stop, … sometimes the colleague in that rush is stressed and you get there, “wait a minute, stay a little bit here… I go there to help the colleague to do the work … so he can take a breath … we are helping each other a lot! (UCL04)

I have used the care I do the OAC (Observation and Behavioral Approach) I have applied with colleagues. (UCL19)

~~this is continuous, because you cannot enter any of the sectors without PPE, if all the clothing is worn ... we are always keeping an eye on our colleague in order to provide protection~~

~~(UCL39)~~

it is at the moment when I saw a colleague, right, having a difficulty, I called him, asked him if he needed anything, if everything was fine with him, and I had that moment of conversation. I believe it has improved the situation he was experiencing at the time! (UCL17)

~~whenever I realized that someone is exposed I try to communicate it because sometimes the person is not doing it wrong; most of the time it’s because they don’t really know (UCL36)~~

we end up looking more at the team, right? I'll look more closely at people's behavior, … realizing who is not well, I call to talk, ... but we end up helping each other more (UCL07)

we are always sometimes a colleague, sometimes we don’t even forget, so we really guide. (UCL15)

**7) DIFFICULTY IN APPLYING “BECOMING AWARE”, IN SPITE OF REALIZING THE IMPORTANCE OF IT**

-I think it would be excellent if we were able to actually apply it because … it is very busy due to the demand … we do not have time to apply the training, but I remember that when I did it it, it was very relaxing, very wonderful (UCL19)

-…it is during working hours I haven't been able to do it, but before leaving home I always stop for a while, I think, I try to breathe, calm down in order to have a more peaceful journey (UCL34)

-… at the beginning I practiced a lot, it was really good it works, but now at this moment it's so busy, that there's no time for us to stop a little bit, …now there's no time for even drinking water, I practiced a lot but now it's all over, but it was very good. (UCL15)

**8) FOCUS FOR LIFE**

-I would say that I even became a better person, because from the moment you take things more clearly, more calmly, you become a better person, so it has helped a lot, not only at work, in life. (UCL24)

-My perception is that it helps a lot in our life, I even put it here in my room I always do it in the morning before leaving home (UCL07)
